# Oral rhabdomyosarcoma: A review

**DOI:** 10.4317/jced.50926

**Published:** 2012-12-01

**Authors:** Ankita Tandon, Kanika Sethi, Anand Pratap Singh

**Affiliations:** 1 Assistant Professor, Department of Oral Pathology & Microbiology, Faculty of Dentistry, Jamia Millia Islamia, New Delhi, India; 2 Senior Lecturer, Department of Oral Pathology & Microbiology, Indraprastha Dental, College & Hospital, New Delhi, India; 3 Senior Lecturer, Department of Oral Medicine & Radiology, Rungta College of Dental Sciences and Research, Kohka-Kurud Road, Bhilai-Durg, India

## Abstract

Rhabdomyosarcoma (RMS) is a rare malignant soft tissue neoplasm comprised of cells derived from the primitive mesen¬chyme. About 35% of RMS arises in the head and neck, are are classified as parameningeal and non-parameningeal forms. These are the most common soft tissue sarcoma of the children, adolescents and young adults. Their etiopathogenesis and its molecular relevance have been emphasized. The first line of treatment is radical excision and this is usually supplemented by radiotherapy. It is believed that adjunct combination chemotherapy may greatly improve the prognosis. Inadequately treated tumours grow in an infiltrative manner and recur in a high percentage of cases. Bone does not constitute an effective barrier to the growth of the tumour and bone invasion is a frequent finding in head and neck rhabdomyosarcomas.

** Key words:**Rhabdomyosarcomas, botryoid, spindle, alveolar, sarcomas, undifferentiated.

## Introduction

Sarcomas are rare, malignant tumors that can arise from mesenchymal tissues at any body site. The histopatho-logic spectrum of sarcomas is broad, presumably because the embryonic mesenchymal cells from which they originate have the capacity to mature into striated skeletal and smooth muscle, adipose and fibrous tissue, bone, and cartilage (1).

Rhabdomyosarcoma was initially described by Weber in 1854 (2). The first published example of rhabdomyosarcoma, the malignancy of striated muscle, was probably a tongue lesion reported in 1854 (3). It is the most common soft tissue sarcoma of the children, adolescents and young adults. They are classified histologically into Embryonal, Botryoid, Alveolar and Pleomorphic varieties, and tend to occur predominantly in three regions: the head and neck, genitourinary tract, and upper and lower extremities (4).

Rhabdomyosarcoma may be defined as a malignant tumor of the rhabdomyoblasts with a microscopic picture simulating that of striated muscle cells (5). Although an uncommon lesion, this tumor is among the most com-mon head and neck cancers in young persons (3).

Rhabdomyosarcoma (RMS) is the most common soft-tissue sarcoma in pediatric patients and accounts for nearly 20% of soft-tissue sarcomas overall. In children, close to 50% of rhabdomyosarcomas arise in the head and neck, most commonly in the parameningeal region, orbit, oral cavity, nasopharynx, sinuses, ear, and neck (3).

## Etiopathogenesis

Skeletal muscle fiber regeneration is a phenomenon that recapitulates several steps of muscle embryogenesis. Originally located in close contact with the myofibre, inside the myofibril basement membrane coating (so-called endomysium tube), satellite cells play a crucial role in this process since they function as myogenic stem cells (6).

Rhabdomyosarcoma is considered to result from malignant change of primitive mesenchymal cells rather than differentiated muscle (7). These primitive mesenchymal tissues exhibit a tendency toward myogenic differentiation and probably originate from satellite cells associated with the embryogenesis of skeletal muscle. Rhabdomyosarcomas (RMS) are thought to originate from immature cells that are destined to form striated skeletal muscle; however, these tumors can arise in locations where skeletal muscle is not typically found (eg, the urinary bladder). Undifferentiated sarcomas (UDS) derive from mesenchyme that cannot be ascribed to a specific tissue lineage (1).

No clear etiologic factors have been identified to account for the occurrence of these malignant neoplastic growths.There is, however, increasing evidence that gene abnormalities may play a role in the development of some childhood malignancies, especially rhabdomyosarcoma(8). Cytogenetic and molecular studies have identified chromosomal translocations and mutations in oncogenes (2).

Commitment and maintenance of skeletal muscle differentiation during normal skeletal muscle myogenesis, as well as in Rhabdomyosarcoma, is regulated by a family of closely related genes (eg. myoD1, myogenin, myf-5, MRF-4), termed the MyoD family. These genes encode a series of DNA-binding proteins that control the activation and transcription of genes encoding muscle enzymes and proteins, such as creatine kinase and desmin. Of these genes, myoD1 and myf-5 most likely contribute to the determination of the myogenic state; whereas myogenin includes terminal differentiation and MRF-4 is thought to be involved in the maintenance of the mature adult muscle phenotype. MyoD1 and myogenin are considered useful markers in diagnosing Rhabdomyosarcomas and for differentiating it from other soft tissue tumors (9).

## Molecular Pathology

Molecular pathology to classification of RMS may help to solve the controversies in RMS subtypes by the use of objective criteria based on genetic differences. It has been described that patients with the variant PAX7-FKHR translocation have a more favorable prognosis and there has been a strikingly better outcome in patients with metastatic disease and variant-trans¬location-positive alveolar RMS (estimated 4-year overall survival, 75% vs. 8% for patients with PAX3-FKHR-positive alveolar RMS) (10).

Myogenin is considered a sensitive and specific marker for RMS and this is thought to be more specific than desmin and muscle-specific actin and more sensitive than myoglobin (11). Myogenin and desmin are sensitive and specific immunohistochemical markers for head and neck RMS. Other immunohistochemical markers have been used to help with the difficult diagnosis of RMS, including myogenic nuclear regulatory proteins such as MyoD1 and myogenin that act as transcription factors and stimulate myogenesis. MyoD1 is a marker of the myoid lineage which is expressed by fetal myoblasts and is important for the transition from cell proliferation to differentiation. A variety of differentiated cell types can be converted into skeletal muscle after transfection with MyoD1 through the activation of muscle-specific genes. Myogenin is a myoid differentiation marker. In this respect, it has been reported that the loss of normal proliferation and differentiation control may theoretically lead to the formation of RMS (2).

Genetic abnormalities have been demonstrated in rhabdomyosarcomas and implicated in the pathogenesis of rhabdomyosarcoma. One line of genetic study is on the tumor suppressor gene, p53. Rhabdomyosarcoma is the basic cancer type of a familial cancer syndrome, the Li-Fraumeni syndrome (12). In Li-Fraumeni syndrome families, there is a germline p53 mutation (13). It is speculated that Li-Fraumeni p53 mutant alleles may be weak alleles that lack dominant negative activity and can be tolerated and passed in the germline of families. In nonfamilial, sporadic cases of rhabdomyosarcoma, there is a high frequency and diversity of p53 mutation(14). P53 gene is located on the short arm of chromosome 17 (17p). The wild type p53 controls cell proliferation at a G1/S checkpoint in the cell cycle and the mutated type presumably loses this regulatory function so that the affected tissue undergoes tumorous growth (14). Second line of genetic study on rhabdomyosarcoma concerns the loss of heterozygosity of the short arm of chromosome 11 (llp), which occurs in embryonal rhabdomyosarcomas. It is presumed that a recessive tumor suppressor gene, the Rb gene, resides in the region 11 p 15.5-terminal. Loss of heterozygosity with inactivation of the paternal Rb gene, that is, by DNA methylation or other events, would lead to tumorous growth. The third line of study regards translocation between the long arms of chromosomes 2 and 13, designated as t(2;13) (q35;q14) (12). This type of cytogenetic abnormality is mainly found in alveolar rhabdomyosarcoma. The translocation results in fusion of a transcription regulator gene, the PAX3, with a transcription factor gene, the ALV, near the translocation site of chromosome 2. This fusion in turn presumably results in transcriptional deregulation and tumorigenesis. Besides these three lines of study, activation and mutation of oncogenes, particularly the RAS oncogenes, have been demonstrated in rhabdomyosarcomas (8).

Antibodies against muscle-specific intermediate filament desmin, muscle-specific actin, and myoglobin provide additional information in the diagnosis of Rhabdomyosarcomas but may lack sensitivity and specificity. Extensive myogenesis studies have revealed the existence of a family of genes encoding a series of DNA-binding proteins that play a critical role in the commitment of mesenchymal progenitor cells to the myogenic lineage and in their subsequent differentiation (9).

Alveolar rhabdomyosarcoma has been demonstrated to have a characteristic translocation (2;13)(q35;q14)) fusing the PAX3 gene with the FKHR gene. The PAX3 gene is believed to regulate transcription during early neuromuscular development and FKHR is a member of the forkhead family of transcription factors. It is hypot-hesized that the fusion transcription factor inappropriately activates transcription of genes that contribute to a transformed phenotype. Genomic amplification of such genes as MYCN, MDM2, CDK4 and PAX7-FKHR is mainly a feature of the alveolar subtype. Loss of alleles and imprinting at 11p15.5 together with disruption of genes such as IGF2 have also been implicated in rhabdomyosarcoma development. Activation of IGF2 seems to be a feature of all rhabdomyosarcomas, even though 11p15.5 loss of heterozygosity and IGF2 loss of imprinting are alterations predominantly associated with the embryonal and alveolar subtypes, respectively. The ALK gene is located at 2p23 and encodes a novel transmembrane receptor tyrosine kinase, ALK, belonging to the insulin receptor family of tyrosine kinase. This protein is expressed in normal tissue during embryogenesis but is absent from all types of adult tissue, except for scant weakly positive cells in the central nervous system, in scattered neurones, glial cells and endothelial cells. NPM-ALK chimeric protein is thought to be up-regulated as a result of the t(2;5) as classically seen in anaplastic large cell lymphoma, resulting in an up-regulated mitogenic signal through aberrant phosphorylation of intracellular substrates (15).

Embryonal RMS is associated with loss of heterozygosity (LOH) at the 11p15 locus, which affects the expres-sion of insulinlike growth factor (IGF), a growth factor of RMS. p16INK4A (p16) gene induces dephosphoryla-tion of pRB by inhibiting binding of cyclin-dependent kinase (CDK)4 and CDK6 to cyclin D, resulting in G1 growth arrest. The discovery that p16 gene is mutated or deleted in a striking proportion of human tumors raised the possibility that abnormalities in p16 might predispose to cancer development (16).

In contrast to alveolar neoplasms, embryonal RMS rarely bear this genetic lesion. In embryonal tumors where the above noted translocations are not seen, LOH at the 11p15 locus is almost uniformly identified. This region of the short arm of chromosome 11 contains a number of imprinted genes implicated in oncogenesis, including H19, IGF2 and p57KIP2; however, the relevant genetic target of this putative loss-of-function mutation has yet to be identified. The 11p15 region is imprinted in normal tissue, with only the paternal allele being transcribed (15).

Myostatin, a member of the TGF-b superfamily, is a key regulator of skeletal muscle growth and development. Myostatin has been shown to inhibit the proliferation of normal myoblasts by altering the levels and activity of members of the cell cycle machinery. Specifically, in response to myostatin treatment, the cyclin-dependent kinase inhibitor p21 is upregulated and the level of Cdk2 is slightly reduced resulting in a loss of cyclin-E–Cdk2 activity. This loss of activity results in the loss of Rb phosphorylation causing cell cycle arrest (14).

S Charrasse et al have analysed cadherin-mediated cell adhesion in RMS, nonepithelial tumors of skeletal muscle origin, and found multiple defects in the expression of cadherins and catenins in RMS-derived cell lines. Different mechanisms might be involved in this decrease in cadherin and catenin expression. In particular, the altered expression and/or activity of transcription factors, which regulate the promoters of the adherens junction proteins, might cause these expression defects. Slug and Snail, two zinc-finger transcription factors, decrease E-cadherin promoter activity in cancers. Additionally, CpG hypermethylation of the E-cadherin promoter is an important mechanism of E-cadherin gene inactivation(17).

AKT plays a critical role in controlling the balance between cell survival and apoptosis. RMS cell lines that express high level of phospho-AKT, at least RH30 and SMS-CTR, seem to depend on AKT pathway for cell proliferation and survival (18,19).

## Clinical appearance

The incidence of RMS is highest in children 1-4 years of age, falling to a lower rate at 10-14 years, and remai-ning steady between 15-19 years of age (10). It is rare after 45 years of age (3). A slight preference for males has been reported, with these tumors mainly occurring in the first and second decades of life (2).

The presenting signs and symptoms of RMS are variable, and are influenced by the site of origin, the age of the patient, and the presence or absence of distant metastases (1). The signs and symptoms may include pain, pa-resthesia, loss of teeth and trismus as a result of factors such as advanced tumor stage, infiltrative growth and tumor location (2). A history of antecedent trauma is uncommon, and fever is only rarely present (1).

The head and neck are the most frequently affected regions, followed by the orbit (35% of cases), trunk and extremities, intra-abdominal organs and genitourinary tract (23%) (2). Site predilection in the oral cavity varies according to diffe¬rent authors, some finding that the palate is the commonest site, while others report that the tongue is the most common site (10). There is general agreement that the orbit is the most commonly affected site. The nasopharynx and middle ear regions follow in frequency although at least one major study suggests that the soft tissue of the neck may be the second most common site (7). The parameningeal sites, which include nasopharynx, nasal cavity, paranasal sinuses, pterygopalatine, and infratemporal fossa and middle ear, have been associated with extension directly to the central nervous system (CNS) in 35% of cases within 1 year of diagnosis (7). ( Table 1)

**Table 1:**
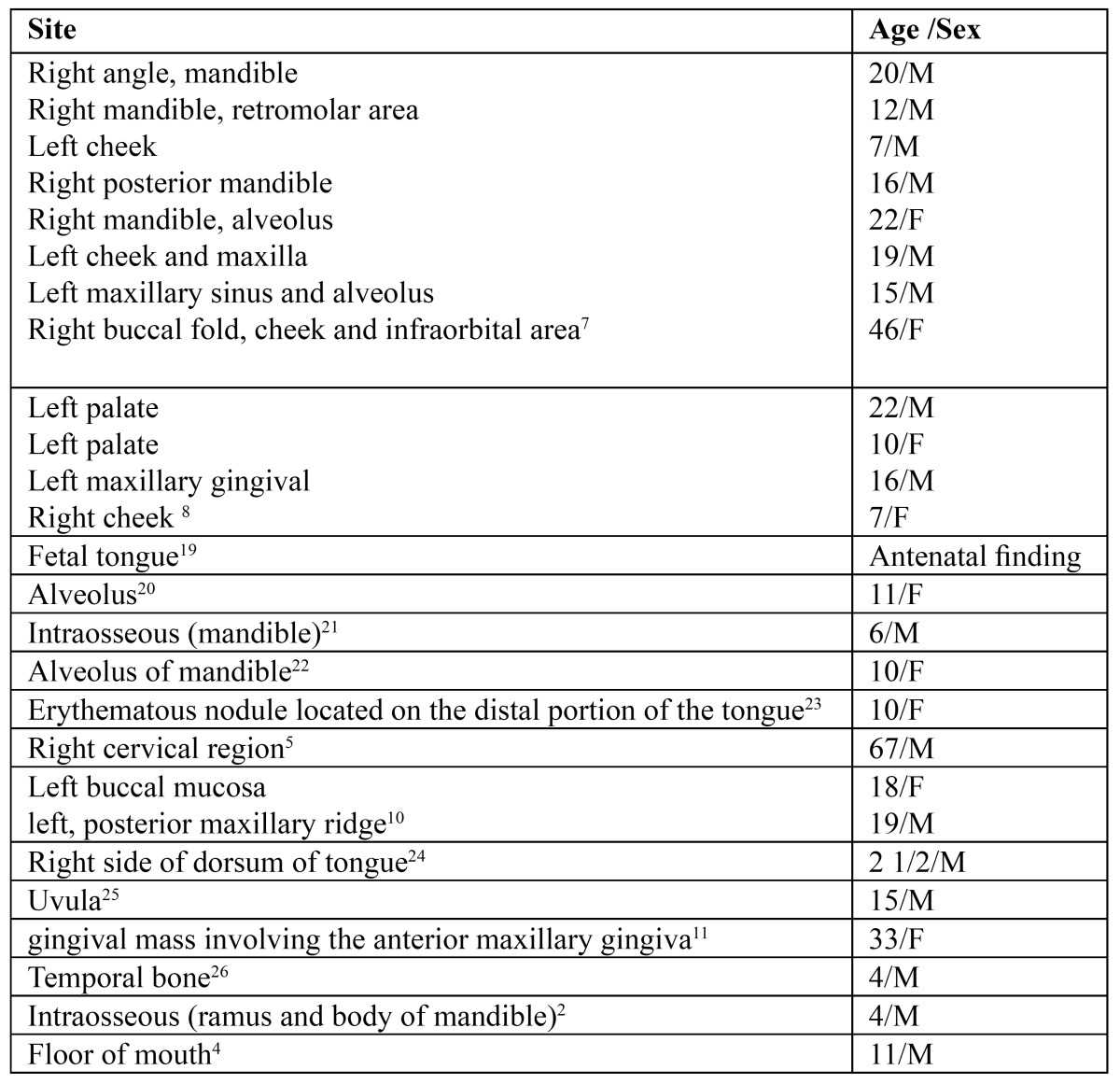
A few reported cases of Rhabdomyosarcoma at diverse locations.

The clinical appearance may exhibit smooth or lobulated surface, sometimes botryoid or grape cluster–like in appearance and definitely becomes fixed to surrounding tissues at an early stage (3).

30% of all head and neck RMS have their origin in intraoral and pharyngeal structures. In affected patients, the tumor expands and infiltrates the muscle from which it arises, presenting first as a well demarcated nodule or polypoid lesion with a soft or gummy consistency. When these lesions grow rapidly they may cause dyspnea, dysphagia, and cough, including acute respiratory obstruction (23).

Initial symptoms may be vague, and may mimic other childhood and adolescent, soft-tissue sarcomas such as fibrosarcoma, leiomyosarcoma and neu¬rofibrosarcoma (10). Of the sarcomas involving the oral cavity in chil-dren, rhabdomyosarcoma, alveolar soft-part sarcoma, fibrosarcoma, leiomyosarcoma, and Kaposi sarcoma are the most common (20).

However, neuroblastoma, another small cell tumor characterized by a diffuse pattern of small round cells and the presence of rosettes/pseudorosettes with pale eosinophilic material is quite similar to the alveolar variant of rhabdomyosarcoma. The frequently elevated level of urinary catecholamines in neuroblastoma aids in the differential diagnosis (20-22).

The differential diagnosis also includes vascular malformations, within which the most common affecting the pediatric airway is the lymphatic or lymphatic-venous malformation (LM). Lymphatic malformations are typically described as cystic lesions present at birth that grow in proportion to the child. Although most head and neck LM are present at birth, they may not become clinically apparent until a child develops an upper respiratory infection or sustains trauma to the area (23).

## Diagnosis

It is possible to detect intraoral mass (RMS tongue) by antenatal ultrasound scan and it warrants a careful perinatal multidisciplinary team approach. The treatment of RMS is site-specific and multidisciplinary efforts offer best results. An initial complete primary resection of tumor with negative gross and microscopic margins is ideal, if possible. Another option is initial incisional biopsy followed by neoadjuvant therapy, to preserve the organ and secondary excision if required. As the sole radiation modality, fractionated high-dose-rate (F–HDR) brachytherapy achieved excellent local control and disease free survival, in properly selected children with soft tissue sarcomas, while preserving normal bones and organ development (24).

Hemophagocytosis is the latest diagnostic clue to monocytic-histiocytic proliferative disorders and are also frequently observed in other hematologic malignancies. Its occurrence in solid tumors is rare, but has been reported in small-cell lung carcinoma, breast carcinoma, medulloblastoma, hemangioendotheliosarcoma and recently, rhabdomyosarcoma (12).

Computed tomography and magnetic resonance imaging (MRI) with contrast are helpful in generating a diffe-rential diagnosis for the child with cystic lesions of the head and neck. These give complementary information regarding the bony and soft tissue extension of the lesion (23).

## Types

There are predominantly three types of Rhabdomyosarcomas:

1. Embryonal variant: Accounts for approximately 49% of all rhabdomyosarcomas and affects mostly children younger than 10 years of age, but it also occurs in adolescents and young adults. It is rare in patients older than 40 years of age.

2. Alveolar variant: It accounts for almost 30% of all rhabdomyosarcomas and tends to arise in patients of age group 10-25 years. It has predilection for deep soft tissues of the extremities. The tumor may arise at other places also though they are rare.

3. Pleomorphic variant: This is a rare variant which almost arises in adults older than 45 years. They arise mostly in the deep soft tissues of the extremities (4).

Enzinger and Weiss proposed two histogenetic possibilities for rhabdomyosarcoma: (a) primitive and undifferentiated mesenchyme origin and (b) embryonal muscular tissue origin (24-26).

The histopathological and molecular spectrum manifested by RMS has led to many classification systems. Horn and Enterline(mid 1900s) divided rhabdomyosarcomas into embryonal (ERMS), alveolar, botryoid, and pleomorphic subtypes. In 1994, histological and biological studies have resulted in the International Classification of RMS and hence four broad subtypes of RMS were established: (a) botryoid and spindle cell RMS (both less common variants of ERMS); (b) embryonal RMS, generally having a superior prognosis; (c) alveolar (including the solid-alveolar variant) RMS, generally having a poorer prognosis and (d) undiffe-rentiated sarcoma, also generally having a poorer prognosis. Finally, a category of sarcoma nor otherwise specified was created for tumors that could not be classified into a speci¬fic subtype. Recently it was added to this classification a subtype of RMS with rhabdoid-like features, whose prog¬nosis is not presently valuable. It is now apparent that rhabdomyosarcoma comprises a group of morphologically similar but biologically diverse lesions (10).

## Histopathologic spectrum

Majority of oral rhabdomyosarcomas are of embryonal type, with small, round or oval tumor cells resembling embryonal or developing voluntary muscle cells. These cells have a finely granular eosinophilic cytoplasm with few cells demonstrating fasciculation or cross-striations. There often is a fibrillar material imparting a clear zone around the nucleus, and the nucleus itself is typically enlarged. On the other hand, more well-differentiated tumors demonstrate elongated, strap shaped or tadpole-shaped rhabdomyoblasts. Occasional giant cells with enlarged or multiple nuclei can also be seen. Mitotic figures are often seen and may be abnormal. The background stroma is scanty and consists of moderately loose to dense fibrous tissue. A background of poorly differentiated ovoid mesenchymal cells is frequently noted, and myxoid zones are commonly seen in the stroma (3).

Histologically, rhabdomyosarcomas were classified by Horn and Enterline into four subtypes: pleomorphic, alveolar, embryonal, and botryoid. However, botryoid rhabdomyosarcoma is a descriptive term for a rhab-domyosarcoma with polypoid or grape-like gross appearance and is considered a variant of the embryonal form. The more differentiated cells are spindle-shaped or strap shaped. Longitudinal and occasionally cross striations can be identified. Binucleation is common, and nuclei are frequently vesicular (8).

The definitive diagnosis is based on histological evidence of myogenesis in the tumor, including giant or multinucleated myoblasts, strap or tadpole cells, and individual tumor cells with or without cross-striations and densely eosinophillic cytoplasm. However, as rhabdomyosarcomas tend to display a spectrum of wide histological features and is often poorly differentiated, its diagnosis is often difficult (9).

Alveolar RMS is typically comprised of small round densely packed cells, arranged around spaces resembling pulmonary alveoli. A solid variant form of alveolar RMS has been identified, which consists of small round densely packed cells without the characteristic alveolar spaces. There does not appear to be any prognostic significance of the solid alveolar variant. Alveolar tumors harbor a distinguishing chromosomal translocation marker, typically t(2;13)(q35;q14). Pleomorphic RMS is currently rarely diagnosed and is characterized by large aggregates of anaplastic cells (15).

Histological classification has prognostic significance. For instance, the botryoid and spindle cell variants have a favourable prognosis, while embryonal rhabdomyosarcomas tend to have a better prognosis than alveolar rhabdomyosarcoma. It is therefore important to classify these tumours correctly (12).

## Treatment

In the advanced stages of the disease, because of the in¬filtrative growth, and depending on the site of the tumor, pain, paresthesia, loosening of the teeth, and trismus occur (10).

Treatment therefore is by a multidisciplinary approach. It consists of surgical removal of the tumor followed by multiagent chemotherapy with or without radiotherapy since RMS tend to metastasize to bone marrow, bone marrow aspiration should be a part of the staging procedure (4,5,11). Total tumor resection confers the most favorable treatment outcome. However, due to its invasive nature complete tumor resection is often difficult (5). Prognosis remains poor for patients with metastatic disease at presentation and poor response to chemotherapy. Radiotherapy is targeted at the pre-chemotherapy sites of disease. Chemoradiation following complete surgical resection appears to be the ideal therapy (5). Multimodal approaches combining surgical resection, radiation, and systemic chemotherapy need further investigation(1,5).

Metastasis may develop during the course of illness in approximately 20% of the cases with major sites being the lung, lymph node and bone marrow followed by heart, brain, meninges, pancreas, liver and kidney. Lungs are involved in nearly two thirds of patients of metastasis (4,5).

Oral rhabdomyosarcoma favourably is treated by radical surgical excision followed by multiagent chemotherapy, usually a combination of vincristine, dactinomycin, and cyclophosphamide. If complete resection is not possible then postoperative radiotherapy may be employed. Five-year survival rates have improved dramatically from less than 10% before the 1960s to 65% today. Stage I lesions have an even better prognosis (80%). Metastasis, when it occurs, is via either blood or lymphatic vessels, usually to cervical lymph nodes, lungs, bones, or brain (3).

ERMSs are treated with multimodality therapy and have a favorable prognosis relative to other subtypes of RMS, with classic ERMS having a 66% 5-year survival rate and botryoid and well-differentiated spindled variants having a nearly 90% 5-year survival rate. Adverse prognostic factors in ERMS include adult age, parameningeal location, head and neck location, large size, and unresectability (3).

Complete resection with histologically free margins is often unfeasible without exenteration, but chemotherapy and radiotherapy enable complete remission in the majority of cases, so mutilating surgery is currently reserved only for unresponsive tumours. Unfortunately, important radiotherapy-induced late sequelae (functional changes, ie., cataract, orbital hypoplasia, facial asymmetry) are common (18).

## Prognostic relevance

Oral rhabdomyosarcomas are classified within the non-parameningeal group of tumors, which do not tend to invade the central nervous system (10). As a result of their aggressive neoplastic behavior characterized by immature and highly invasive cells, RMS are associated with high rates of recurrence and generalized metastases through the hematogenic and/or lymphatic routes(2). With the advent of combined surgical, chemo-therapeutic, and radiotherapeutic management of RMS, the five-year survival rate is approximately 85% for this RMS subtype (10).
